# Aerobic high‐intensity interval training and maximal strength training in patients with unspecific musculoskeletal disorders improve V̇O_2peak_ and maximal strength more than moderate training

**DOI:** 10.1002/ejsc.12126

**Published:** 2024-05-15

**Authors:** Håkon Hov, Geir Eithun, Eivind Wang, Jan Helgerud

**Affiliations:** ^1^ Treningsklinikken Medical Rehabilitation Clinic Trondheim Norway; ^2^ Faculty of Health Sciences and Social Care Molde University College Molde Norway; ^3^ Department of Circulation and Medical Imaging Faculty of Medicine and Health Sciences Norwegian University of Science and Technology Trondheim Norway; ^4^ Unicare Hokksund Medical Rehabilitation Centre Hokksund Norway; ^5^ Department of Psychosis and Rehabilitation Psychiatry Clinic St. Olavs University Hospital Trondheim Norway

**Keywords:** exercise, fibromyalgia, low back pain, maximal oxygen uptake, resistance training

## Abstract

Improving peak oxygen uptake (V̇O_2peak_) and maximal strength are key objectives of rehabilitation for patients with unspecific musculoskeletal disorders (MSDs). Although high‐intensity training yield superior outcomes for these factors, patients with MSDs may not tolerate high‐intensity due to pain and fear. Therefore, we examined the effect and feasibility of incorporating aerobic high‐intensity intervals (HIITs) and maximal strength training (MST) in a standard clinical rehabilitation program for patients with unspecific MSDs. 73 patients (45 ± 10 years) with MSDs partaking in a standard, public, and 4‐week rehabilitation program were randomized to high‐intensity training (HG: 4 × 4 minutes intervals at ∼90% of maximal heart rate; HR_max_, and 4 × 4 repetitions leg press at ∼90% of 1 repetition maximum; 1RM, with maximal intended velocity) or keep todays treatment of low‐to moderate‐intensity training (MG: various cycling, walking, and/or running activities at ∼70%–80% of HR_max_ and 3 × 8 − 10 repetitions leg press at ∼75% of 1RM without maximal intended velocity). HG improved V̇O_2peak_ (12 ± 7%) and leg press 1RM (43 ± 34%) more than moderate‐intensity group (V̇O_2peak_; 5 ± 6%, 1RM; 19 ± 18%, both *p* < 0.001). We observed that no adverse events and no between‐group differences in dropout rate or self‐reported quality of life (both *p* > 0.05). There were positive correlations between improved V̇O_2peak_ and improved physical (*p* = 0.024) and emotional (0.016) role functioning. We conclude that both high‐intensity interval training and MST are feasible and improve V̇O_2peak_ and maximal strength more than standard low‐to moderate‐intensity treatment of patients with unspecific MSDs. Our findings suggest that high‐intensity training should be implemented as a part of standard clinical care of this patient population.

## Highlights

- Implementing aerobic high‐intensity interval training (HIIT) and maximal strength training (MST) induce a twofold effect on V̇O_2peak_ and 1 repetition maximum compared to standard public short‐term rehabilitation in patients with unspecific musculoskeletal disorders (MSDs).
- Concurrent HIIT and MST are as feasible as moderate exercise for patients with unspecific MSDs and should be implemented as a component in standard clinical care for these patients.
- An improved V̇O_2peak_ is associated with improved physical and emotional role functioning.

## 1 INTRODUCTION

Peak oxygen uptake (V̇O_2peak_) and maximal skeletal muscle strength are the two pillars of physical function and health (Booth et al., 2012; Schaap et al., 2012) and strongly associated with longevity (Harb et al., 2019; Ruiz et al., 2008). Rehabilitation and treatment and applying exercise as medicine, should therefore pursue training modalities and formats that optimizes the effect on these key components. However, some patient populations may suffer from pain‐ and fear‐inducing conditions that hampers compliance to effective training protocols, for example, low back pain and fibromyalgia (Pocovi et al., 2023; Vancampfort et al., 2023). Indeed, such indications have been forwarded with regards to the application of aerobic and strength training with high‐intensity (Vancampfort et al., 2023).

Aerobic high‐intensity interval (HIIT), carried out at ∼90% of maximal heart rate (HR_max_), is well documented to induce greater improvements in V̇O_2peak_ compared to moderate training with ∼70%–80% of HR_max_ in healthy individuals (Helgerud et al., 2007) as well as a wide range of patient populations, for example, substance‐use disorders (Flemmen et al., 2014), peripheral arterial disease (Slørdahl et al., 2005), and coronary artery disease (Rognmo et al., 2004). Similarly, maximal strength training (MST), carried out as few repetitions with heavy loads at ∼90% of 1 repetition maximum (1RM), with maximal intended velocity in the concentric phase, is shown to induce greater increases in maximal strength compared to conventional strength training at low or moderate intensity (∼70% of 1RM) in healthy (Heggelund et al., 2013) and diseased individuals, for example, peripheral arterial disease (Wang et al., 2010), schizophrenia (Nygård et al., 2021), and Parkinson's disease (Helgerud et al., 2020). In addition to being crucial for primary prevention of lifestyle‐related diseases (Booth et al., 2012), aerobic power and muscle strength are often positively associated with self‐reported quality of life (Haglo et al., 2022; Oscar García López et al., 2016), suggesting that these factors are of both objective and subjective importance for most patient populations.

Musculoskeletal disorders (MSDs) encompass several diagnoses, including diseases which may be objectively observed, such as osteoporosis and osteoarthritis, and less objectively identifiable diseases, such as unspecific low back pain and fibromyalgia (Smith et al., 2014). For all MSDs, pain and its consequences, for example, reduced participation in society, work, social life, and sports, are common traits (Blyth et al., 2019). Therefore, it is conceivable that patients with unspecific MSDs may associate high‐intensity exercise with pain, and consequently hesitate to engage in such exercises (Palstam et al., 2016; Pocovi et al., 2023). Yet, contrary to these common beliefs, laboratory trials have demonstrated that both HIIT and (relatively) high‐intensity strength training seem to be both safe and feasible for patients with various unspecific MSDs (Atan et al., 2020; Cerini et al., 2022; Verbrugghe et al., 2019; Vilarino et al., 2023). However, it is worth noting that there is a lack of studies applying MST (i.e., ≥90% 1RM) in this population. Moreover, there are contradicting findings or low quality of evidence for any differences in subjective health‐related outcomes and compliance when applying high‐intensity training compared to more conventional treatments or moderate exercise in patients with fibromyalgia (Atan et al., 2020; Vilarino et al., 2023) and chronic low back pain (Cerini et al., 2022; Verbrugghe et al., 2019).

Given that high‐intensity training appears to be safe and effective for patients with unspecific MSDs in controlled laboratory investigations, and that this population may be more reluctant to conduct such exercises because of fear and pain‐avoidance, we sought to examine the effect and feasibility of implementing HIIT and MST in a standard, public, and short‐term clinical rehabilitation program. We compared the responses of maximal leg press strength and V̇O_2peak_ following a rehabilitation program, including today's treatment, which mainly comprises of low‐ and moderate‐intensity exercises, to a modified version applying high‐intensity exercises. We hypothesized that the high‐intensity group (HG) would improve maximal strength and V̇O_2peak_ more than the moderate‐intensity group (MG), and that both exercise regimes would be feasible for the patients with unspecific MSDs, with no differences in compliance nor quality of life.

## 2 METHODS

### 2.1 Participants

**FIGURE 1:**
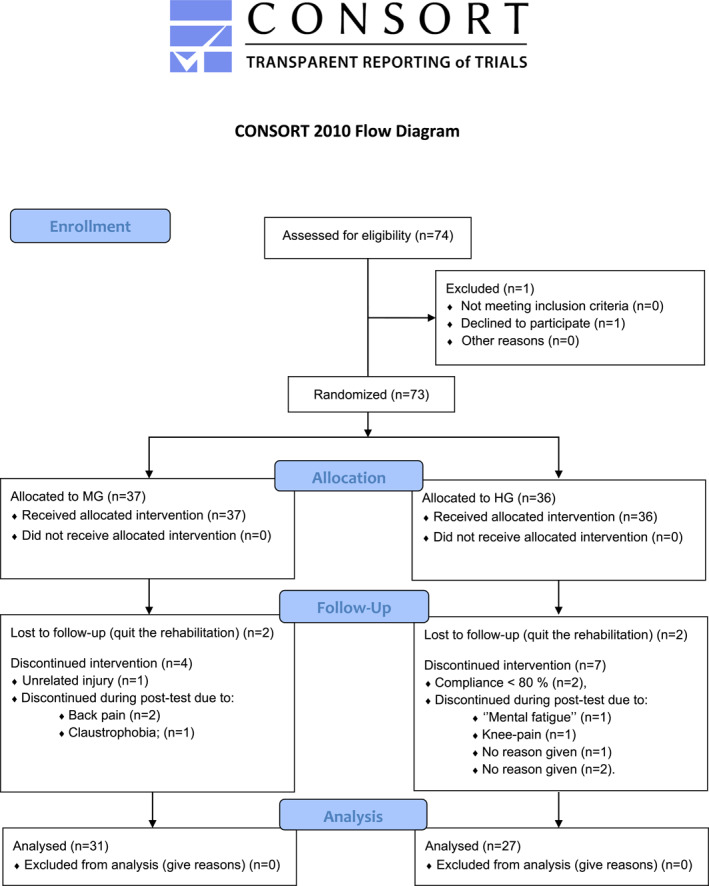
CONSORT flow diagram of the study.

Seventy‐four patients with MSDs were invited to participate in the current study while participating in a 4‐week rehabilitation program. Seventy‐three gave their informed written consent and were subsequently randomized to either HG or MG (Figure 1). In general, the patients were not engaged in regular physical exercise, though most had previously been advised by a general practitioner to conduct physical activities, such as walking or cycling. Patients included in the rehabilitation program had MSDs of unspecific cause. Unspecific low back pain and unspecific multilocational pain (including fibromyalgia) were the most common causes of referral to the rehabilitation. Some patients included had specific MSDs, for example, rheumatoid arthritis, albeit such diagnoses were never the main cause of their pain and disability, as assessed by at least two medical doctors and a multidisciplinary team. Given that unspecific origins of pain and disability were a prerequisite for partaking in this rehabilitation program, detailed characteristics of concrete diagnoses are not presented. Patient characteristics are given in Table 1.

**TABLE 1:** Patient characteristics at baseline.

	High‐intensity group (*n* = 36)	Moderate‐intensity group (*n* = 37)
Age (years)	44 ± 10	45 ± 11
Body mass (kg)	79.2 ± 14.4	86.6 ± 20.3
Body height (cm)	171 ± 11	171 ± 8
BMI (kg height^−2^)	27.5 ± 5.6	29.8 ± 5.6
V̇O_2peak_ (mL kg^−1^ min^−1^)	34.0 ± 7.9	33.8 ± 7.0
Sex (male/female)	12/24	15/22
Diagnoses^a^		
Unspecific back pain *n*, (%)	11 (31%)	19 (51%)
Unspecific neck pain *n*, (%)	6 (17%)	1 (3%)
Multisite unspecific pain *n*, (%)	17 (47%)	14 (38%)
Inflammatory rheumatic disease *n*, (%)	2 (6%)	2 (5%)
Verified skeletal disorder *n*, (%)	5 (14%)	2 (5%)
Depression *n*, (%)	3 (8%)	7 (19%)

*Note*: Data are presented as mean ± SD and diagnoses are presented as absolute numbers and as a percentage within each group.

Abbreviations: BMI, body mass index; VO_2peak_, peak oxygen uptake.

^a^ Patients with more than one relevant diagnosis are counted several times.

All patients included in the rehabilitation program were invited to partake in this study. Thus, inclusion criteria for the rehabilitation program and the current study were the same. Patients had to be on sick leave or unemployed with long‐lasting MSDs (˃3 months), referred by a general practitioner or hospital doctor, and assessed and found eligible for participating by a multidisciplinary team at the rehabilitation center. Undecided applications for disability pension or insurance, severe psychiatric disorders, and unsettled medical investigations were exclusion criteria for admittance to rehabilitation. Patients were excluded if they withdrew from one or more test‐procedure or had a compliance of less than 80%. The study was conducted in accordance with the Declaration of Helsinki and approved by the Regional Committee for Medical Research Ethics.

### 2.2 The rehabilitation program

The rehabilitation program lasted for 4 weeks, with a 6‐h schedule, and 5 days a week. The multidisciplinary team consisted of physicians, nurses, physical therapists, social workers, and sports educators. Besides the strength and endurance training sessions (see below), the rehabilitation program was identical for HG and MG and included group activities with low‐moderate intensity, such as yoga, stretching, walking, resistance training with body weight, rubber bands or balls, aqua exercises, body awareness, relaxation, cognitive behavioral modification, and education. The aim of the standard rehabilitation program was to improve the patients' physical function and reduce their pain‐related barriers for participating in society, work, and social settings.

### 2.3 Training interventions

The strength and aerobic endurance interventions were led by a physical therapist and/or a sports educator. Upper body strength training of ∼75% of 1RM (treatment as usual) were included for both groups. Only leg press was applied as strength training for the lower extremities (see the respective group descriptions). Two minutes of rest interspersed all sets of strength training for both groups. The aerobic exercise modalities were spinning and walking or running with an incline both on a treadmill and outdoors, and the distribution of these modalities were equal between groups. A total of 10 strength sessions and 15 aerobic endurance sessions were administered during the 4‐week program for each group.

#### 2.3.1 Moderate‐intensity training group (MG)

The strength training applied in the horizontal leg press was 3 sets of 8–10 repetitions at ∼75% of 1RM. MG had no particular focus on maximal mobilization in the concentric phase. Patients were regularly encouraged to increase training load, but there were no strict rules for when a patient had to increase the weights. MG had no systematic focus on intensity during the aerobic endurance trainings except for one weekly HIIT on a spinning bike. This single weekly HIIT session consisted of 4–5 intervals of 4–5 min duration interspersed by 3 min of active recovery guided by subjective feelings of effort and shortness of breath.

#### 2.3.2 High‐intensity training group (HG)

The strength training in the horizontal leg press consisted of 4 sets of 4 repetitions at ∼90% of 1RM and is referred to as maximal strength training; MST. Emphasis was placed on maximal intended velocity and mobilization in the concentric phase (Behm et al., 1993), and the eccentric phase was performed with relatively slow velocity to minimize eccentric forces (Haglo et al., 2022). When a patient was able to conduct more than four repetitions in the last set, the load was increased by 4.5 kg in the following session. The aerobic endurance trainings for HG were HIIT guided and controlled by HR. This was conducted as 4 intervals of 4 min duration, aiming to elicit 85%–95% of HR_max_, interspersed by 3 min of active recovery at an intensity corresponding to ∼70% of HR_max_ (Helgerud et al., 2007).

### 2.4 Physiological testing procedures

All tests were completed in the same order before and after the intervention.

Blood pressure was measured in a standardized sitting position after 3 min of rest with a half automatic blood pressure device (Bosu‐Medicus, Bosch + Sohn GMBH U. CO., Germany, and cuff TYP CA01). The arm was in a relaxed position with light flexion in the elbow, and the cuff was fixed 1–2 cm proximal to the elbow.

Maximal leg press strength was tested using a horizontal leg press machine (Technogym 451, Italy). A default position was standardized individually, yet all with a knee joint angle of approximately 90°. All positioning and settings of the body and apparatus were noted for each individual, assuring similar procedures during pre and posttest. A warm‐up series of 10 repetitions on a submaximal level was applied. Leg press 1RM was measured by repeated lifts, separated by 1‐min breaks, with loads increasing by 4.5 kg until failure.

Walking economy and V̇O_2peak_ were tested at a motorized treadmill (h/p/cosmos T 150 med). A Cortex Metamax II (Cortex Biophysik GmbH) was used for measures of pulmonary gas exchange, and HR was monitored using Polar RS400 (Polar Electro). A 10‐min warm‐up at approximately 50%–60% of HR_max_ preceded the walking economy test. Walking economy was measured at 5% incline and 3.0 km h^−1^ for 4 min. Mean V̇O_2_ during the last 60 s was defined as walking economy with visual inspection to assure steady state was achieved. The graded exercise test to measure V̇O_2peak_ started directly following the walking economy test. The workload (speed and/or incline) was increased every minute until volitional exhaustion despite strong verbal encouragement. V̇O_2peak_ was defined as the highest 30‐s average V̇O_2_.

### 2.5 Quality of life questionnaire

In a health‐related quality of life questionnaire, Norwegian RAND‐36 (SF‐36) was given to the patients before and after rehabilitation (Ware et al., 1992). SF‐36 is a non‐disease dependent questionnaire, which evaluates eight dimensions on a score from 0 to 100: Physical functioning, bodily pain, physical role functioning, general health, vitality, emotional well‐being, social functioning, and emotional role functioning. For all eight dimensions, a higher score represents a better self‐reported quality of life or health outcome.

### 2.6 Statistical analyses

Statistical analyses were conducted using IBM SPSS Statistics 29 software (IBM Corp.). Differences between groups were examined by two‐way ANOVA, and data of V̇O_2peak_ and 1RM were tested for normality using QQ‐plots and the Shapiro–Wilk test. Assumptions of normal distribution and equal variances were met. The SF‐36 data was also analyzed by two‐way ANOVA, and bootstrapping was additionally performed and contrasted to the results from ANOVA. Dropout rates between groups were analyzed with Fisher's exact test. Relationships between data from physiological tests and SF‐36 were explored using Spearman's correlation test. *p* < 0.05 was taken as the level of significance in the statistical tests. Results are presented as mean ± standard error in figures and mean ± standard deviation in text and tables.

## 3 RESULTS

### 3.1 Dropouts and compliance

58 patients completed all training (>80%) and testing according to protocol, and there were no differences in dropout (*p* = 0.386) or compliance (*p* = 0.229) between groups. Reasons for dropouts are reported in Figure 1. We did not observe any differences between the dropouts (*n* = 15) and the non‐dropouts (*n* = 58) when analyzing baseline physiological factors or characteristics of diagnoses.

### 3.2 Physiological factors

**FIGURE 2:**
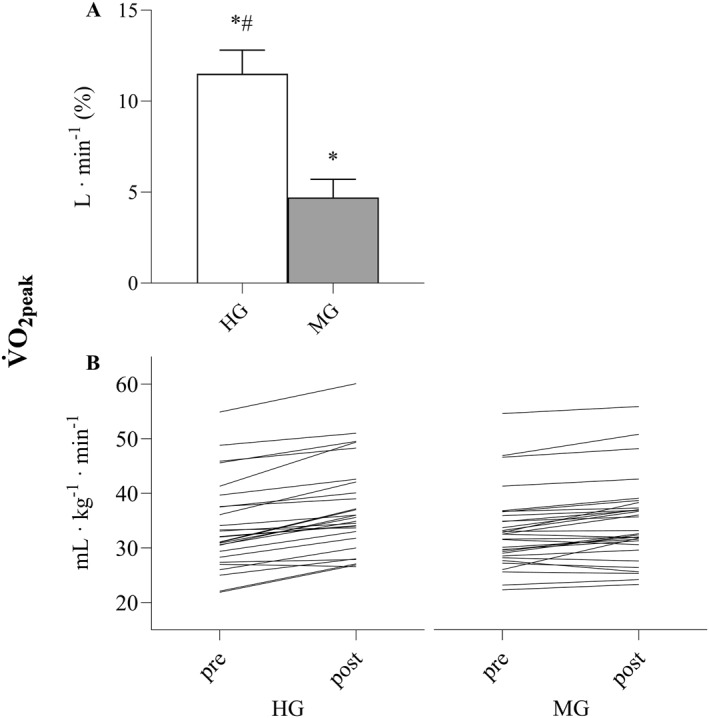
(A): Percentage change in peak oxygen uptake (V̇O_2peak_) for the high‐intensity training group (HG) and moderate‐intensity training group (MG). (B): Individual V̇O_2peak_‐values pre and post HG and MG. Significant difference from pre to post within group *p < 0.001, between groups #*p* < 0.001.

**FIGURE 3:**
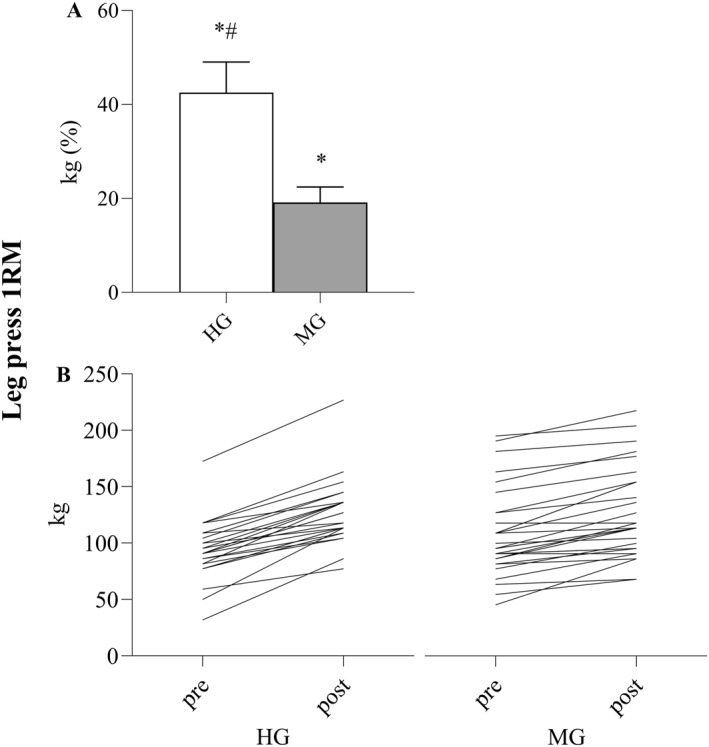
(A): Percentage change in leg press one repetition maximum (1RM) for the high‐intensity training group (HG) and moderate‐intensity training group (MG). (B): Individual 1RM‐values pre and post HG and MG. Significant difference from pre to post within group *p < 0.001, between groups #*p* < 0.001.

HG improved V̇O_2peak_ (Figure 2) and leg press 1RM (Figure 3) more than MG (both *p* < 0.001). No other differences between the groups were observed, and there were no differences between groups at baseline (Table 2).

**TABLE 2:** Physiological data before and after interventions.

	High‐intensity group (*n* = 27)			Moderate‐intensity group (*n* = 31)			Main effect (time)	Time × group
	Pre	Post	Pct Δ (Hedges' g)	Pre	Post	Pct Δ (Hedges' g)	*p*‐value (*η*_p_^2^)	*p*‐value (*η*_p_^2^)
V̇O_2peak_								
L min^−1^	2.65 ± 0.64	2.94 ± 0.66***	11.5 ± 6.8 (1.72)	2.87 ± 0.75	3.01 ± 0.80***	4.7 ± 5.8 (0.77)	<0.001 (0.625)	<0.001 (0.179)
mL kg^−1^ min^−1^	33.8 ± 8.1	37.3 ± 8.4***	10.7 ± 6.9 (1.64)	33.0 ± 7.0	34.6 ± 7.5***	4.8 ± 6.1 (0.84)	<0.001 (0.636)	<0.001 (0.193)
Walking economy								
V̇O_2_ (L min^−1^)	1.31 ± 0.27	1.19 ± 0.25***	9.1 ± 7.9 (1.07)	1.37 ± 0.31	1.25 ± 0.30***	8.1 ± 10.3 (0.82)	<0.001 (0.400)	0.745 (0.002)
V̇O_2_ (mL kg^−1^ min^−1^)	16.5 ± 1.9	14.8 ± 1.7***	9.7 ± 7.8 (1.16)	15.6 ± 2.0	14.3 ± 1.4***	7.9 ± 10.7 (0.74)	<0.001 (0.410)	0.536 (0.007)
HR (beat min^−1^)	127 ± 12	114 ± 12***	10.2 ± 7.3 (1.30)	123 ± 16	112 ± 13***	8.0 ± 7.2 (0.99)	<0.001 (0.586)	0.260 (0.023)
1RM leg press (kg)	92.9 ± 25.6	127.2 ± 28.0***	42.5 ± 33.6 (2.44)	108.0 ± 37.7	125.8 ± 39.1***	19.1 ± 18.3 (1.33)	<0.001 (0.797)	<0.001 (0.280)
Resting blood pressure								
Systolic (mmHg)	134 ± 15	128 ± 12**	4.4 ± 7.1 (0.60)	135 ± 16	129 ± 11**	3.7 ± 9.7 (0.45)	<0.001 (0.222)	0.895 (0.000)
Diastolic (mmHg)	82 ± 8	77 ± 7***	6.7 ± 7.5 (0.83)	82 ± 10	78 ± 8***	4.2 ± 5.8 (0.71)	<0.001 (0.398)	0.221 (0.027)
Body mass (kg)	79.6 ± 14.5	80.2 ± 14.5*	0.7 ± 1.5 (0.46)	88.3 ± 21.3	88.2 ± 20.8	0.0 ± 2.0 (0.09)	0.359 (0.015)	0.081 (0.053)
BMI (kg height^−2^)	27.6 ± 5.5	27.8 ± 5.5*	0.7 ± 1.5 (0.46)	30.0 ± 5.8	30.0 ± 5.6	0.0 ± 2.0 (0.07)	0.296 (0.019)	0.094 (0.049)

*Note*: Data are presented as mean ± SD. Walking economy measured at 3 km h^−1^ and 5 % elevation on a treadmill.

Abbreviations: 1RM, one‐repetition maximum; BMI, body mass index; HR, heart rate; Pct, Δ; percentage, change; VO2peak, peak oxygen uptake; *η*_p_^2^, partial eta squared.

Significant difference from pre to post within group *p ≤ 0.05; **p ≤ 0.01; and ***p ≤ 0.001.

### 3.3 Health‐related quality of life questionnaire, SF‐36

No differences in SF‐36 data were observed between groups neither at baseline nor in response to the interventions (Table 3). With MG and HG pooled (*n* = 58), there were positive correlations between the change in V̇O_2peak_ and the change in emotional (rho = 0.33 and *p* = 0.016) and physical (rho = 0.31 and *p* = 0.024) role functioning. Bootstrapping did not change the results from the parametric analysis of the SF‐36 data.

**TABLE 3:** Health‐related quality of life before and after interventions.

	High‐intensity group (*n* = 27)			Moderate‐intensity group (*n* = 31)			Main effect	Time × group
	Pre	Post	Hedges' g	Pre	Post	Hedges' g	*p*‐value (*η*_p_^2^)	*p*‐value (*η*_p_^2^)
Physical functioning	68 ± 20	77 ± 19*	0.51	64 ± 21	77 ± 19***	0.86	<0.001 (0.333)	0.323 (0.020)
Bodily pain	28 ± 15	48 ± 17***	1.11	31 ± 20	51 ± 21***	0.78	<0.001 (0.473)	0.932 (0.000)
Physical role functioning	16 ± 27	54 ± 35***	1.11	17 ± 30	40 ± 39*	0.46	<0.001 (0.357)	0.207 (0.032)
General health	54 ± 18	66 ± 20***	0.75	51 ± 17	67 ± 15***	0.92	<0.001 (0.435)	0.334 (0.019)
Vitality	36 ± 17	53 ± 20**	0.57	35 ± 21	57 ± 20***	1.05	<0.001 (0.395)	0.418 (0.013)
Emotional well‐being	59 ± 21	76 ± 12***	0.85	62 ± 15	77 ± 13***	0.99	<0.001 (0.474)	0.605 (0.005)
Social functioning	52 ± 30	68 ± 20*	0.48	55 ± 21	69 ± 23**	0.57	<0.001 (0.224)	0.790 (0.001)
Emotional role functioning	36 ± 37	73 ± 36***	0.81	37 ± 41	62 ± 35**	0.50	<0.001 (0.319)	0.329 (0.019)

*Note*: Data are presented as mean ± SD.

Abbreviation: *η*_p_^2^, partial eta squared.

Significant change from baseline within group * *p* ≤ 0.05; **p ≤ 0.01; and ***p ≤ 0.001.

## 4 DISCUSSION

Maximal strength and V̇O_2peak_ are the two pillars of physical health and functioning (Booth et al., 2012; Schaap et al., 2012), and high‐intensity training has previously been documented to induce superior improvements in these factors when compared to moderate‐intensity training in healthy individuals and patient populations less limited by pain and fear (Brobakken et al., 2019; Helgerud et al., 2007; Slørdahl et al., 2005). Yet, patients with unspecific MSDs may be opposed to conduct high‐intensity training because of pain and fear‐avoidance (Pocovi et al., 2023; Vancampfort et al., 2023), possibly resulting in low compliance in a real‐world setting, and consequently blunted effects on physiological outcomes. Therefore, we compared the effects of high‐versus moderate‐intensity training on maximal strength, V̇O_2peak_, feasibility, and self‐reported health‐related quality of life in a standard, public, and short‐term rehabilitation program. Our main findings were that HG did improve V̇O_2peak_ and maximal leg press strength more than MG, while both programs were similarly feasible in terms of dropout rates and compliance. Thus, our findings demonstrate that rehabilitation for patients with unspecific MSDs should implement both HIIT and MST.

### 4.1 High‐intensity intervals and MSDs

The current study demonstrated that patients in HG improved V̇O_2peak_ more than those in MG. This finding is in accordance with our hypothesis, and the vast body of literature advocating that HIIT more effectively increase V̇O_2peak_ compared to moderate‐intensity training both in healthy subjects (Helgerud et al., 2007) and various patient groups (Rognmo et al., 2004; Støa et al., 2017), including low back pain (Verbrugghe et al., 2019). However, it is important to notice that HIIT in the current study had a high *aerobic* intensity. A high aerobic intensity ensures that the cardiac output of the heart is targeted (Wang et al., 2014), and such intervals are recently documented to be superior to intervals with even higher overall intensity both in terms of increasing V̇O_2max_ and safety (Helgerud et al., 2023; Hov et al., 2023). In contrast to our findings, there are previous reports of similar efficacy of high‐ and moderate‐intensity training in patients with fibromyalgia (Atan et al., 2020). A possible reasons for this discrepancy between studies is that Atan and Karavelioğlu (Atan et al., 2020) applied a somewhat lower exercise intensity (>80% of HR_max_) in their HIIT‐group, yet a sufficiently high aerobic intensity is crucial to effectively overload the oxygen transport system and induce superior improvements compared to moderate continuous training (Helgerud et al., 2007; Wenger et al., 1986). Also, the patients in Atan and Karavelioğlu had a much lower baseline V̇O_2peak_ (∼19 mL min^−1^ kg^−1^) compared to our study (Atan et al., 2020), possibly affecting comparisons between groups since exercise‐induced adaptations in V̇O_2peak_ are related to initial training status (Wang et al., 2014; Wenger et al., 1986).

### 4.2 Maximal strength training and MSDs

Our findings show that leg press MST improved 1RM more than moderate‐intensity strength training without maximal intended velocity in patients with unspecific MSDs. This is in accordance with our hypothesis, previous research on other patient populations (Helgerud et al., 2020; Mosti et al., 2011), and healthy subjects (Heggelund et al., 2013). Strength training applied for patients with unspecific MSDs is previously often described as “high‐intensity,” albeit the intensity applied in such studies is rarely above 80% of 1RM (Kristensen et al., 2012; Verbrugghe et al., 2019; Vilarino et al., 2023). In line with this notion, the intensity of ∼75% of 1RM in MG in the current study corresponds to what is often described as “high‐intensity”. To the best of our knowledge, this is the first study to apply MST, that is, ∼90% 1RM, in this population, demonstrating an additional effect of ∼90% compared to ∼75% of 1RM in patients with unspecific MSDs. MST with emphasis on maximal intended velocity in the concentric phase is designed to target the nervous system in particular, and effectively improves maximal strength, rate of force development, and efferent neural drive (Fimland et al., 2010; Tøien et al., 2021; Unhjem et al., 2021).

### 4.3 The feasibility of high‐intensity exercise for patients with unspecific MSDs

There was no difference in dropout rate between MG and HG, indicating that HIIT and MST are similarly feasible as today's treatment with low‐to moderate‐intensity. This is in accordance with our hypothesis and several studies describing their experimental group as “high‐intensity” (Cerini et al., 2022; Verbrugghe et al., 2019). In fact, recent studies in patients with inflammatory rheumatic disease, which, in a similar fashion to the current study population, are characterized by pain and stiffness, also reported that high‐intensity training was feasible (Haglo et al., 2021, 2022). These observations contrast a recent meta‐analysis that reported an increased dropout from high‐intensity compared to lower‐intensity exercise in patients with fibromyalgia (Vancampfort et al., 2023). Yet, this meta‐analysis presented a trim‐and‐fill adjusted dropout rate of 19.2% across 122 exercise interventions for patients with fibromyalgia (Vancampfort et al., 2023), while another meta‐analysis demonstrated a dropout rate of 17.4% across 11 RCTs of sham versus manual treatment for low back pain (Lavazza et al., 2021). These dropout rates reported in meta‐analyses are comparable to the 20.5% dropout rate in our study, indicating that high‐intensity exercise is as feasible as low‐ and moderate‐intensity exercises or passive treatment modalities. It is in this context important to note that the patients in our study were partaking in a rehabilitation program for 6‐h per day and therefore, received substantial support from both health‐care professionals and each other. For example, they were reassured that muscle soreness is a normal and harmless response when they experienced this after pretests and initial training sessions. We cannot know how this support affected compliance, but it is reasonable to assume less adherence to exercise if the patients were to apply the exercise protocols without initial help and reassurance.

### 4.4 Health‐related quality of life for patients with MSDs

Both HG and MG improved all eight dimensions of self‐reported health‐related quality of life following the interventions with no differences between groups. The improved SF‐36 scores in our study agrees with previous studies of exercise‐interventions for musculoskeletal pain (Kayo et al., 2012; Pieber et al., 2014). Our findings show that both moderate‐ and high‐intensity training improve self‐reported quality of life, including improved pain scores, indicating that the fear of aggravated pain induced by high‐intensity exercises may be exaggerated in populations with unspecific MSDs. Moreover, the change in two of the SF‐36 dimensions, physical and emotional role functioning, positively correlated with the change in V̇O_2peak_. Together, these results indicate that concurrent HIIT and MST is not only feasible for patients with unspecific MSDs but also a potent treatment for their subjective physical and emotional health.

### 4.5 Limitations

First, the uncontrolled and unstructured nature of today's treatment limits the generalizability and replicability of the exercise‐interventions in MG. Anecdotally, our observations confirm that the approximate intensities provided, that is, ∼75% of 1RM and ∼70%–80% HR_max_, reflects the actual intensities applied. Second, we must highlight that this study was an intense short‐term intervention of only 4 weeks. Therefore, we are not able to present any long‐term effects of the protocols. Third, we have no analysis of the cost‐benefit ratio of our interventions. However, there are no reasons to assume a different cost of applying MG and HG, indicating that the cost‐benefit ratio of HG is likely superior for physiological factors. Of note, implementation of a 3‐week intensive rehabilitation after hospital discharge have been demonstrated to yield better quality of life and lower medical costs after 1‐year for patients with arthritis compared to usual care with weekly follow‐up (Bulthuis et al., 2008).

### 4.6 Clinical implications

We demonstrate that in 4 weeks, HIIT and MST induce about a twofold increase in both V̇O_2peak_ and maximal strength compared to standard rehabilitation applying moderate‐intensity training in patients with unspecific MSDs. No differences in self‐reported quality of life or feasibility were observed. As for many other patient populations, we do not observe any general diagnosis‐specific reasons to reduce the intensity of training for patients with unspecific MSDs. Importantly, MST is considered safe due to the execution of movement. While the concentric phase is performed with heavy loading and maximal intended velocity, the potentially more harmful eccentric phase is performed in a slow and controlled fashion to minimize risk of muscle, tendon, and joint damage (Tøien et al., 2018). Previously, MST has been investigated in frail patient populations (e.g., patients with inflammatory rheumatic disease (Haglo et al., 2022), patients undergoing hip surgery (Berg et al., 2021), peripheral arterial disease (Wang et al., 2010), and patients with Parkinson's disease (Helgerud et al., 2020)) and older adults (Berg et al., 2018). Similarly, HIIT is documented to be safe and provides excellent results to patient populations at risk for cardiovascular events (Helgerud et al., 2009; Rognmo et al., 2004; Wisløff et al., 2007). Individual responses to exercise vary (Figures 2B and 3B), which can be reflected by the 10th and 90th percentiles of adaptations in HG (1RM; 16% and 76% and V̇O_2peak_; 3% and 20%) and MG (1RM; 0% and 38% and V̇O_2peak_; −2% and 12%), and clinicians should be aware of this variability. The general benefits of exhibiting relatively high V̇O_2peak_ and muscle strength are great, as these physical factors are strongly associated with disease prevention (Pedersen et al., 2015), physical function (Booth et al., 2012; Schaap et al., 2012), and survival (Harb et al., 2019; Ruiz et al., 2008). Therefore, we recommend that patients with unspecific MSDs should engage in high‐intensity training if their objective is to maximize combined improvements in physical health, function, and quality of life through increased V̇O_2peak_ and maximal strength.

## 5 CONCLUSION

Substituting today's treatment of moderate‐intensity endurance and strength training with aerobic HIIT and maximal strength training results in twice as large improvements of V̇O_2peak_ and maximal strength following a short‐term rehabilitation program. Since moderate‐ and high‐intensity were similarly feasible, high‐intensity training should be advocated for adults with unspecific MSDs if the aim is to improve V̇O_2peak_ or maximal strength.

## CONFLICT OF INTEREST STATEMENT

The authors declare that they have no conflicts of interest.

## Data Availability

The data that support the findings of this study are available from the corresponding author upon reasonable request.
